# Assessment of the effect of mindfulness monotherapy on sexual dysfunction symptoms and sex-related quality of life in women

**DOI:** 10.1093/sexmed/qfad022

**Published:** 2023-06-05

**Authors:** Izabela Jąderek, Katarzyna Obarska, Michał Lew-Starowicz

**Affiliations:** Department of Psychiatry, Centre of Postgraduate Medical Education, Warsaw 01-813, Poland; Institute of Psychology, Polish Academy of Sciences, Warsaw 00-378, Poland; Department of Psychiatry, Centre of Postgraduate Medical Education, Warsaw 01-813, Poland

**Keywords:** mindfulness, female sexual dysfunctions, sexual therapy

## Abstract

**Background:**

Mindfulness-based therapies (MBTs) are frequently used in the treatment of sexual dysfunctions. So far, there has not been sufficient evidence for the effectiveness of interventions based on mindfulness monotherapy.

**Aim:**

The aim of the study was to assess the effect of mindfulness monotherapy on the reduction of sexual dysfunction symptoms and sex-related quality of life.

**Methods:**

We conducted 4 weeks of MBT for 2 groups of heterosexual females: 1 with psychogenic sexual dysfunction (WSD) and 1 with no sexual dysfunction (NSD). Overall 93 women were recruited for the study. We collected data via an online survey regarding sexual satisfaction, sexual dysfunctions, and mindfulness-related features at baseline, 1 week after MBT, and follow-up 12 weeks after MBT. Research tools included the Female Sexual Function Index, Five Facet Mindfulness Questionnaire, and Sexual Satisfaction Questionnaire.

**Outcomes:**

Participating in the mindfulness program had a positive effect on women with and without sexual dysfunction.

**Results:**

The overall risk for sexual dysfunction decreased from 90.6% at baseline to 46.7% at follow-up in the WSD group and from 32.5% at baseline to 6.9% at follow-up in the NSD group. Participants in the WSD group reported a significant increase in levels of sexual desire, arousal, lubrication, and orgasm between measurements, although not in the pain domain. Participants in the NSD group reported a significant increase in the level of sexual desire between measurements but not in levels of arousal, lubrication, orgasm, and pain. A significant increase in sex-related quality of life was observed in both groups.

**Clinical Implications:**

The results of the study have a chance to translate into an introduction of a new therapeutic program for specialists and more effective help offered to women experiencing sexual dysfunctions.

**Strengths and Limitations:**

This mindfulness monotherapy research project, which included assessment of meditation “homework,” is the first to verify the potential of MBT in reducing symptoms of psychogenic sexual dysfunctions among heterosexual females. Major limitations include the lack of randomization, an adequate control group, and a validated measure of sexual distress.

**Conclusion:**

The applied training was beneficial in the treatment of sexual dysfunctions in terms of increasing desire and arousal as well as the ability to reach orgasm. However, this approach needs more investigation before it can be recommended in the treatment of sexual dysfunction. The study should be replicated under a more rigorous research design, including adequate control groups and random allocation of participants to study conditions.

## Introduction

The contemporary conceptualization of problems related to the sexual functioning of women, as well as the therapeutic procedures, is based on an integrated biopsychosocial model of sexual activity. The model indicates that biological/organic, psychological, and/or sociocultural factors individually influence the sexual response in women and remain in constant dynamic interaction, affecting the entirety of sexual activity.^1^ The following have been mentioned among the psychogenic factors associated with a negative impact on female sexual activity^2^: anxiety and depressed mood^3^; low self-esteem and related intrusive thoughts^4^; stress, fatigue, distraction, and diversion during sexual activity^5^; a focus on performance, a tendency toward excessive self-control, an experience of guilt and shame related to sexual activity, complexes, and a critical attitude toward the body^6^^,^^7^; and negative experiences, including sexual abuse.^8^

### Sexual function and sexual satisfaction

According to Basson and her circular model of sexual response, female sexual function and sexual satisfaction proceed in a complex manner and are affected by numerous psychological issues.^9^ Although many women may experience spontaneous desire, usually the desire for emotional closeness motivates them to participate in sexual activity, and when the sexual desire emerges, they are motivated to continue the activity. Feelings of intimacy and connection with a partner may influence female sexual satisfaction as well as relationship satisfaction, both of which can lead women to be receptive to the next sexual experience.^10^ A large body of research has been conducted to examine the links between sexual desire and sexual satisfaction.^11^ The links are quite clear among partnered individuals: higher sexual desire toward the current partner is associated with other sexual responses and reactions and, moreover, greater sexual satisfaction. Also, partners with high dyadic sexual desire and activity report high sexual satisfaction and optimal psychological functioning.^12^

Sexual satisfaction appears to be an important subject of research.^13^ It is a subjective global judgment of the quality of one’s sexual life; it consists of various components; and it is usually defined as “an affective response arising from one’s subjective evaluation of the positive and negative dimensions associated with one’s sexual relationship.”^14^ Sexual satisfaction is one of the indicators of sexual health and is associated with relationship satisfaction.^13^ Pascoal et al identified 2 aspects of sexual satisfaction: (1) personal sexual well-being, which focuses on pleasure, positive feelings, sexual openness, and sexual response, and (2) dyadic processes, which underlie mutuality, the expression of feelings, creativity, the acting out of desires, and the frequency of sexual activity.^13^ Mutual pleasure plays a crucial role in sexual satisfaction and comes from positive sexual experiences. The findings of various research suggest that sexual satisfaction influences relationship stability and general quality of life.^15^

### Sexual satisfaction and sexual quality of life

In our study, we have investigated the sexual quality of life, which is a broader term than sexual satisfaction and is defined as “the individual’s subjective evaluation of the positive and negative aspects of one’s sexual relationship, and his/her subsequent affective response to this evaluation.”^16^ It also refers to the perception of sexual functions and predicts the consequences of sexual problems as well as mental and physical health.^17^ A desirable level of sexual quality of life is important in sexual and reproductive health and is associated with improvements in general quality of life. Sexual quality of life consists of a few components: psychosexual feelings (anger, worry of partner’s hurt or rejection), sexual and relationship satisfaction (enjoyment, feeling good about oneself), self-worthlessness (feeling like less of a woman, feeling of guilt), and sexual repression (loss of pleasure, avoiding).^18^

### Development and treatment of sexual dysfunctions

Numerous scientific reports indicate the complexity of psychogenic causes of female sexual dysfunctions, determining the need to develop and adapt different psychotherapeutic strategies.^19^

Cognitive-behavioral techniques proved to be effective in the treatment of orgasmic disorders and decreased desire and arousal and partly in the treatment of pain disorders; however, the overall effect size is 0.58 across all sexual dysfunctions.^20^ At the same time, cognitive components, such as dysfunctional sexual beliefs, are described as determinants of the lack of desire, and their presence in turn fosters depressive moods. Fear of intimacy, concentration on oneself and one’s thoughts, and so-called spectatoring, instead of experiencing intimacy with a partner, contribute to the lack of response to sexual stimulation and emotional disconnection from the sexual relationship.^10^ In the analysis of the causes of sexual difficulties among women, demedicalization and a more careful approach to diagnosis are also postulated, taking into account contextual factors and factors resulting from natural changes in the life cycle that may affect sexual activity.^21^

Protocols used for treating anxiety as the main cause of sexual dysfunction have been proposed by Masters and Johnson^22^ as well as Helen Kaplan.^23^ In addition, anxiety-induced distraction during sexual activity contributed to Masters and Johnson’s development of the “sensate focus” technique.^24^ Sensate focus enables partners to focus on experiencing sensations derived from sexual activity and learning how to be attentive to them to limit the formation of unwanted, intrusive thoughts distancing the person from sexual activity at a given moment.^25^ Intrusive thoughts and other subjectively experienced distractors make it difficult for a woman to focus on sensations flowing from the body as a result of sexual activity; they effect a negative assessment of a specific activity and, as a result, a reduction of subjectively experienced arousal and desire.^26–28^

In assessing the causes of female sexual dysfunctions, more and more attention is being paid to interoception: the woman’s ability to recognize signals from the body.^29^ Low scores on the interoception scales correlate with orgasm disorders, arousal disorders, pain, and sexual dissatisfaction.^30^ The awareness of the signals coming from the body influences a greater correspondence between subjectively perceived arousal and genital response in the form of lubrication.^31^

### Mindfulness-based interventions

Mindfulness refers to a mental condition aiming to focus fully on the present moment in an intentional, conscious, and nonjudgmental manner.^32^ Mindfulness encompasses 2 components: self-regulation of attention and adoption of a particular orientation toward one’s experiences.^33^ Self-regulation of attention refers to nonelaborative observation and awareness of sensations, thoughts, or feelings from moment to moment. It requires the ability to anchor one’s attention on what is occurring and the ability to intentionally switch attention from one aspect of the experience to another.^34^ The elements of mindfulness have proved to be effective against common forms of psychological distress—rumination, anxiety, worry, anger—which involve maladaptive tendencies to avoid, suppress, or overengage with one’s distressing emotions and thoughts.^35^

Women are less likely to recognize signs of their own sexual desire and arousal.^36^ Researchers suggest that being able to experience and name feelings and sensations during desire and arousal can lead to increased feelings of pleasure as well as sexual satisfaction.^37^ Given that sexual function and response, such as desire, are generally conceptualized as emotions with subjective or experiential, physiologic, and behavioral components,^38^ we can suppose that by learning to monitor emotions, bodily sensations, and reactions, the woman’s ability to attend to sexual stimuli will improve and sexual function will increase.^39^ Moreover, mindfulness-based interventions that include training of bringing attention to bodily sensations help increase body awareness and body connection, which may be effective in the treatment of spectatoring and several sexual functioning concerns.^40^^,^^41^

Analysis of the literature on the treatment of sexual dysfunctions with mindfulness shows that, so far, there has not been sufficient evidence for the effectiveness of interventions based on monotherapy.^42^ Monotherapy is conceptualized as a single form of therapy to treat a certain condition; therefore, mindfulness monotherapy could be perceived here as a treatment that consists solely of mindfulness practices such as meditations. Admittedly, in the existing body of research focusing on mindfulness in the treatment of sexual dysfunctions, the researchers concentrated on meditation/mindfulness techniques; however, these were combined with other elements of sexual therapy, relaxation, psychoeducation, and cognitive-behavioral interventions.^43^ Because of that, it is difficult to evaluate specific therapeutic factors and formulate precise conclusions. The changes could be a result of one intervention or from a combination of mindfulness exercises, psychoeducation on sexual functioning, sexuality education, as well as group support.^44^

### Psychological mechanisms of mindfulness interventions

Reports indicate that mindfulness techniques positively influence the regulation of emotions, attention, and body awareness.^45^ Mindfulness-based interventions, currently one of the forms of cognitive-behavioral therapy, have proven efficacy in the treatment of depression, anxiety, and addictions—moreover, constituting a significant complement to other therapeutic models.^46^ Yela et al^47^ identified 3 main factors associated with the mental health benefits of mindfulness practices: self-compassion (viewing challenges as human experiences and taking an attitude of kindness toward oneself), presence of meaning in life (seeing valuable and important things in life and valuable objectives to pursue), and reduction of experiential avoidance (which describes an individual’s ability to “stay with” negative thoughts and emotions). They distinguished “occasional” and “regular meditators” and proved that the higher frequency of at-home meditation practice (ie, practicing between meetings with teacher) plays a crucial role in observing beneficial effects. According to the model of Shapiro et al,^34^ mindfulness meditation effects can be explained by the construct of *reperceiving*, which is a meta-mechanism responsible for mobilizing 4 proximal mechanisms associated with positive health outcomes: values clarification (involves identifying one’s important personal values, which are expected to increase the meaning of life and values-consistent behavior), exposure (ability to “stay with” negative emotions and states), self-regulation (ability to monitor and adapt emotions and behavior), and cognitive-behavioral flexibility (ability to process important available information and produce appropriate and adaptive behavioral responses). Additionally, Vago and Silbersweig indicated “self-transcendence,” which influences mental health and can be conceptualized as “a self-other connection.”^48^

### Mindfulness in the treatment of sexual dysfunctions

The literature to date related to the reduction of symptoms of sexual dysfunction as a result of mindfulness training is currently largely focused on somatic etiology—dysfunctions resulting from such factors as disease or trauma.^42^^,^^49^^,^^50^ Studies have been mostly carried out in small groups and heterogeneous in terms of etiologic dysfunction (eg, as a result of neurologic disease, hysterectomy, removal of the fallopian tubes with the ovaries, and cancer).^51^ In addition, in some of the studies, the participants underwent hormone therapy, which affects the sexual response and makes it difficult to objectively assess the effectiveness of mindfulness interventions.^52^ A recent meta-analysis examined the influence of mindfulness techniques on the reduction of symptoms of sexual dysfunction. It showed that among women who benefited from various interventions in which mindfulness practice was present, an improvement in sexual functioning was observed, with emphasis on subjectively assessed satisfaction and desire. However, pain during intercourse was the domain where improvement was the least frequent, and no increase in physiologic indicators associated with arousal was noted.^42^ The limited number of studies in this area does not allow one to determine the optimal number of mindfulness therapeutic sessions (ie, they differ in terms of number of meetings and duration) or the time between these meetings enabling the desired therapeutic effects to be obtained.^42^ The available studies lack clear information about the qualifications of the person conducting the training and about the suggested mindfulness practices and other interventions; yet, 4 mindfulness sessions proved to be sufficient to achieve the majority of positive results despite the inclusion of various educational elements and therapeutical techniques.^43^^,^^44^ The available results suggest that mindfulness techniques constitute a promising therapeutic method with a range of applications in the treatment of female sexual dysfunctions as well as sexual satisfaction and well-being.^53^

As mentioned, in available research, mindfulness interventions are usually combined with cognitive-behavioral and relaxation techniques and with psychoeducation on sexuality. To evaluate the therapeutic factor of mindfulness, it seems reasonable to prepare a program based solely on mindfulness practice. At the same time, it is worth stressing that the most commonly applicated mindfulness programs—mindfulness-based stress reduction (MBSR) and mindfulness-based cognitive therapy (MBCT)—are a combination of psychoeducation, learning to observe thoughts/emotions, and communication, and they contain elements of short lectures on stress or depression.^54^^,^^55^

### Aim of the study

The study aimed to assess the effect of mindfulness therapy on sexual dysfunction symptoms and sex-related quality of life and the persistence of the effects after its completion. We checked how mindfulness practices can contribute to the improvement of sexual functioning. Practices included developing the ability to be present “here and now,” with full awareness of a specific moment related to feelings, thoughts, and sensations flowing from the body, without judging and without changing one’s thoughts and feelings.

Our hypotheses were as follows:

*Hypothesis 1:* A significant improvement in sexual functioning will be observed among women with sexual dysfunctions after completing the mindfulness program.

*Hypothesis 2:* The improvement in sexual functioning among women with sexual dysfunction will contribute to an increase in sexual satisfaction and sexual quality of life.

*Hypothesis 3:* The effects on sexual functioning and sexual quality of life will persist 3 months after the end of the program.

*Hypothesis 4:* The improvement in sexual quality of life will be observed among women without sexual dysfunctions who practiced mindfulness meditations.

## Method

### Participants

#### Inclusion criteria

Inclusion criteria were as follows. Participants had to be females aged 20 to 45 years. They had to identify themselves as heterosexual, have a permanent sexual partner, and engage in sexual activity yet experience difficulties in sexual functioning. They were further required to meet the diagnostic criteria of a sexual dysfunction of psychogenic etiology for a minimum of 6 months (orgasmic, sexual interest/arousal, or genitopelvic pain/penetration disorder), and they had to report distress due to the experienced dysfunction (hereafter, group with sexual dysfunction [WSD]). The study also included women who undertook sexual activity, did not experience difficulties and distress in sexual functioning, and wanted to learn meditation and check whether it improved their quality of sexual life (hereafter, group with no sexual dysfunction [NSD]). Finally, consent was required to participate in the study.

#### Exclusion criteria

Exclusion criteria included taking medications that may significantly affect the sexual response (eg, antidepressants), participation in group training, current psychotherapy, and sexual therapy or counseling.

### Procedure

#### Recruitment

Recruitment was carried out through advertisements in ambulatory settings where patients experiencing sexual dysfunctions were being consulted, and information was published in the press and on the internet (social networks, internet forums on health and sexuality, and announcements about free events). In addition to a diagnostic history of sexual dysfunction and assessment of personal distress, a general medical history was taken. The women were given information about the study formula and mindfulness training, as well as recommendations for individual practice between meetings. The participants were informed about anonymity, and a summary conversation with the teacher after completing the training was suggested.

#### Diagnostic interview

The diagnostic interview and group mindfulness training were conducted by one clinical sexologist (Izabela Jąderek), who is a certified mindfulness teacher of MBSR and mindfulness-based compassionate living and is qualified for the treatment of sexual dysfunctions as well as the teaching of mindfulness and meditation. The upper age limit (45 years) is associated with the possibility of the onset of menopause.^56^ The lower age limit (20 years) is based on the mean age of sexual initiation among Polish women.^57^ We decided to conduct a diagnostic interview according to criteria specified by the *DSM-5*; therefore, we assessed the level of distress for each participant.

Each participant signed informed consent before entering the study.

#### Materials including mindfulness intervention

Participants completed a battery of online questionnaires before the training, a week after the end of the last meeting, and 12 weeks from the date of the last meeting. Before the training, demographic data were obtained from the participants as well as self-reported measures.

#### Measures

The Female Sexual Function Index (FSFI) is a women’s sexual functioning self-assessment tool. The questionnaire consists of 19 questions, and each item is rated on a scale from 0 to 5 or 1 to 5, allowing the assessment of functioning in the last 4 weeks.^58^^,^^59^ The assessment covers 6 domains: pain related to sexual activity, sexual satisfaction, orgasm, lubrication, arousal, and desire. Higher scores on subscales of the FSFI indicate better functioning in a given sexual area. The questionnaire is available in many languages, and empirical research has confirmed the good psychometric properties and reliability of the test (Cronbach alpha >0.70). The adaptation and validation of the Polish version of the questionnaire proved it to be a good tool for assessing female sexual dysfunctions in Poland,^60^ although it was employed only among heterosexual women.

The Five Facet Mindfulness Questionnaire (FFMQ) is currently the most popular tool for measuring mindfulness. The tool, in keeping with the assumptions of the mindfulness model, measures 5 factors: acting with awareness, nonreactivity, nonjudging, observing, and describing.^61^ The questionnaire, currently used around the world, is a good tool for use in clinical and nonclinical populations. The FFMQ was translated into Polish and subjected to a validation procedure confirming its psychometric abilities.^62^ Reliability values (Cronbach alphas) for individual domains are as follows: nonreactivity = 0.66, observing = 0.73, acting with awareness = 0.79, describing = 0.74, and nonjudging = 0.86. The results of the FFMQ validation studies, as performed in various countries, are consistent with Baer’s research results and the questionnaire’s author and confirm the reliability of the tool.

The Sexual Satisfaction Questionnaire (KSS) is a Polish tool for assessing one’s sexuality.^63^ It is constructed of 10 statements measuring one’s attitude (cognitively and emotionally) toward one’s own sexual activity. The authors created a tool measuring sexual satisfaction in which the operational variables are holistically compatible with a definition of sexual health. The scale can be used by women and men in formal and informal relationships. Individual answers are assigned a score from 0 to 5. The validation tests confirmed the good psychometric properties of the questionnaire and its reliability (Cronbach alpha = 0.83).^64^

After the second and third training sessions, the participants provided information on the frequency of performing individual meditation practice (“homework”) between the meetings. Participants were asked to provide information of the quantity and quality of each meditation presented during meetings (“body scan,” “sitting meditation,” “mindful yoga,” “interoception meditation,” and “sexuality meditation,” as well as short practices such as “3 minutes for mindful breathing”), and they documented how often they practiced each assigned exercise every week. Each item was rated on a scale involving a frequency in terms of days: once a week, twice a week, 3 or 4 times a week, more often, never.

#### Training

Each group included women from the WSD and NSD groups, so none of them could be identified by other group members as experiencing difficulties in sexual functioning; the participants were informed that during the meetings, they were not obliged to talk about their experiences related to sexual activity. Four 2.5-hour meetings were prepared, 1 week apart, consisting only of mindfulness training based on long meditations supplemented with short daily mindfulness practices. Long meditations (45 minutes) were planned, as based on the MBSR program: *body scan meditation*, where participants are taught to pay attention to all sensations coming from the body (starting from the toes); *sitting meditation*, during which participants learn to observe their sensations, emotions, thoughts, and sounds from “here and now” in a benevolent, nonjudgmental, and accepting way; *mindful yoga*, which teaches body awareness^42^; short practices in the form of daily conscious breathing, attentively performed as a single daily activity^65^; and long meditations prepared by the author. For example, the *interoception meditation* focused on learning to recognize and pay attention to various physiologic sensations flowing from the body (eg, hunger, fatigue, desire, arousal), and in the *sexuality meditation*, participants learned to observe their reactions in response to associations and memories related to sexuality (eg, body images, sexual expression, beliefs about closeness, passion, sex, femininity). At the end of each meeting, the moderator presented homework in the form of recommendations for individual practices between meetings: long meditations performed at least 3 or 4 times a week with a recording received from the moderator, as well as a short meditation every day.

### Ethics

The study was approved by the Ethics Committee of the SWPS University (2019) and performed in accordance with the Declaration of Helsinki.

A summary of the study procedure is illustrated in Table 1.

**Table 1:** Study stages.

1. Application addressed to the email address provided in the announcement
2. Taking the diagnostic history online
3. Supplying information about qualifying for participation in the study or the inability to participate. Signing informed consent to participate in the study
4. Questionnaires: first measurement
5. Four mindfulness on-site sessions with the moderator, lasting 2.5 h with an interval of 1 wk, and individual practice following the recommendations provided by the moderator
6. Questionnaires: second measurement—1 wk after the end of MBT intervention
7. Questionnaires: third measurement—12 wk after the end of MBT intervention
8. A summarizing telephone conversation with the moderator

Abbreviation: MBT, mindfulness-based therapy.

### Software and devices

We used an online survey site (SurveyMonkey) to implement and deliver the research procedure to participants, who completed questionnaires via this platform using their personal computers. Women received via email files in MP3 format containing recordings of training sessions intended for listening at home on personal devices (eg, computer, smartphone). All data collected with questionnaires were analyzed with SPSS (version 27.0; IBM) for parametric tests (mostly normality test, Student *t*-test, correlation, and multivariate analysis of variance [MANOVA] and analysis of variance).

### Data analysis

To examine the effect of mindfulness training on sexual functioning and satisfaction with sexual life, we conducted a repeated measures MANOVA. As the outcome variables might be related, we performed a MANOVA with 21 dependent variables—7 dimensions of sexual response (FSFI: lubrication, pain, orgasm, satisfaction, arousal, desire, and overall) at 3 time points (baseline, 1 week after the last training, and follow-up)—and with 1 independent variable (WSD and NSD groups). Then, we conducted post hoc analysis and pairwise comparisons for significant main effects. The Shapiro-Wilk test (for normality of distribution) was performed for the differences between the measurement a week after the intervention and the baseline measurement for all dimensions of mindfulness on the FFMQ, sexual response on the FSFI, and sex-related quality of life on the KSS. If the distribution was not normal, the Spearman coefficient was used in the next step of the linear correlation analysis, and the Pearson coefficient was used for the remaining variables. Analysis of the Student *t*-test for independent samples was carried out to check whether there were differences in the risk of sexual dysfunction and in the level of sexual satisfaction depending on whether the women did their mindfulness practice homework (at least 2 practices a week) or not (≤1 practice).

## Results

### Demographic information

Ninety-three women were recruited for the study: 53 for the WSD group and 40 for the NSD group (Table 2).

**Table 2:** Demographic data.^a^

	**Group, %**		
	**WSD (n = 53)**	**NSD (n = 40)**	***P* value**
Age, y, mean ± SD	30.66 ± 7.43	31.15 ± 7.02	.75
Relationship status			
Married	36.2	35.0	.59
Informal relationship	57.4	60.0	.67
Divorced or separated			—
Single having 1 sexual partner	6.4	5.0	.66
Education level			
Primary			—
Vocational			—
Secondary	21.3	17.5	.47
Higher	78.7	80.0	.55
Academic degree		2.5	—
Residence population			
10 000	6.4	7.5	>.99
>10 000-50 000	8.5	5.0	.41
>50 000-150 000		7.5	—
>150 000-500 000	4.3	17.5	.1
>500 000	80.9	62.5	.1
Are you religious?			
Yes	38.3	32.5	.37
No	61.7	67.5	.79

Abbreviations: NSD, no sexual dysfunction; WSD, with sexual dysfunction.

a Data are presented as percentages unless noted otherwise. Blank cells indicate a data point of 0.

### Participants per group and response rate

The “lost to follow-up” rate—a dropout between the second and third measurements—was 11.37% in the WSD group but only 6.45% in the NSD group (Table 3). From the WSD group, 11 participants did not respond to our messages, and 8 reported the reasons for giving up training: the practice needed too much involvement; the training took too much time; and the practice caused boredom and impatience. In the NSD group, 5 participants did not explain their reason for resignation, and 4 reported difficulties finding time for practice or a change of plans.

**Table 3:** Participants and response rates by measurement.

	**Measurement, No. (%)**		
**Group**	**First**	**Second**	**Third**
WSD	53	34 (64.15)	30 (56.6)
NSD	40	31 (77.5)	29 (72.2)

Abbreviations: NSD, no sexual dysfunction; WSD, with sexual dysfunction.

**Table 4:** Correlations between changes in FFMQ scores from baseline to 1 week after intervention and changes in sexual response (FSFI) and sexual quality of life (KSS).^a^

	**FFMQ, *r* or ρ**									
	**Nonreactivity**		**Observation**		**Aware actions**		**Description**		**Nonjudgmental**	
	**WSD**	**NSD**	**WSD**	**NSD**	**WSD**	**NSD**	**WSD**	**NSD**	**WSD**	**NSD**
Desire	0.27	−0.06	0.11	−0.14	0.09	−0.01	0.21	−0.04	0.24	0.02
Arousal	0.45^**^	−0.07	0.02	0.03	0.23	0.15	0.35^*^	0.18	0.24	−0.02
Lubrication	0.18	−0.14	−0.25	0.03	0.004	0.21	0.35^*^	0.14	0.001	0.3
Orgasm	0.35^*^	−0.13	0.09	−0.19	0.14	0.07	0.23	0.1	0.23	−0.1
Satisfaction	0.21	0.26	0.06	−0.09	0.16	0.08	0.36^*^	0.15	0.26	0.01
Pain	−0.2	−0.09	−0.32	0.01	−0.07	0.1	−0.15	0.06	−0.25	−0.17
Sexual dysfunction	0.34	0.01	0.03	0.01	0.3	0.08	0.36^*^	0.18	0.14	−0.19
Sexual satisfaction	0.46^**^	0.08	0.31	−0.09	0.37^*^	0.28	0.6**	0.26	0.45^**^	0.22

Abbreviations: FFMQ, Five Facet Mindfulness Questionnaire; FSFI, Female Sexual Function Index; KSS, Sexual Satisfaction Questionnaire; NSD, no sexual dysfunction; WSD, with sexual dysfunction.

a Correlation analysis based on *r* (Pearson) and rho (Spearman), depending on normal distribution of variables.

^*^ *P* < .05.

^**^ *P* < .01.

### Associations among mindfulness dimensions, sexual response, and sex-related quality of life

Our research aimed to examine if the change in mindfulness dimensions, sexual response, and sex-related quality of life across time would correlate. Thus, we calculated the difference in scores in each dimension of the variables of interest. Then, we checked if there was a relationship between changes in the mindfulness dimension and sexual functioning.

In the WSD group, the difference in the nonreactivity scores was positively correlated with the difference in arousal, pain, and sexual satisfaction (KSS) scores. The differences in aware-actions and nonjudgmental scores were positively correlated with the difference in sexual satisfaction scores. The difference in the description scores was positively correlated with the difference in arousal, lubrication, satisfaction, and sexual dysfunction risk scores and positively with the difference in sexual satisfaction scores (Table 4).

In the WSD group, the difference in the nonreactivity scores was positively correlated with the difference in the orgasm and sexual satisfaction (KSS) scores. The difference in the observation scores was positively correlated with the difference in the desire and satisfaction scores and positively with sexual satisfaction. The difference in the description scores was positively correlated with the difference in the desire and arousal scores and positively with sexual satisfaction. The difference in the nonjudgmental scores was positively correlated with the difference in sexual satisfaction scores.

In the NSD group, the difference in nonreactivity scores was positively correlated with the difference in sexual satisfaction (KSS) and negatively with pain scores. The differences in the aware-actions and nonjudgmental scores were positively correlated with the difference in the desire scores (Table 5).

### Sexual functioning and dimensions of mindfulness during training

According to the FSFI scores at baseline, 48 (90.56%) women in the WSD group and 13 (32.5%) in the NSD group obtained results below the cutoff, suggesting a high risk of sexual dysfunction. Based on the KSS in the baseline measurement in the WSD group, 79.2% of women assessed their quality of sex life as low, 18.9% as average, and 1.9% as high. In the NSD group, 20% of women reported a low quality of sex life, 10% medium, and 70% high (Tables 6 and 7).

To examine how mindfulness training influences the different dimensions of sexual functioning, we conducted a repeated measures MANOVA for groups with a specific dysfunction based on the cutoff score of FSFI completed at baseline (Table 8).

**Table 5:** Correlations between changes in FFMQ scores from baseline to follow-up and changes in sexual response (FSFI) and sexual quality of life (KSS).^a^

	**FFMQ, *r* or ρ**									
	**Nonreactivity**		**Observation**		**Aware actions**		**Description**		**Nonjudgmental**	
	**WSD**	**NSD**	**WSD**	**NSD**	**WSD**	**NSD**	**WSD**	**NSD**	**WSD**	**NSD**
Desire	0.09	0.33	0.36^*^	0.34	0.13	0.41^*^	0.43^*^	0.28	0.36	0.43^*^
Arousal	0.28	−0.12	0.09	0.16	0.002	0.01	0.42^*^	0.09	0.31	0.14
Lubrication	0.28	−0.01	−0.01	0.33	0.1	0.23	0.14	−0.21	0.24	0.23
Orgasm	0.45^*^	−0.29	0.05	−0.12	0.25	0.03	0.19	−0.29	0.17	0.03
Satisfaction	0.16	0.24	0.39^*^	0.27	0.15	0.12	0.27	0.04	0.37	0.34
Pain	0.14	−0.39^*^	−0.22	−0.19	0.09	0.18	0.01	−0.32	0.19	−0.34
Sexual dysfunction	0.25	−0.11	0.12	0.18	0.07	0.25	0.32	−0.2	0.35	0.27
Sexual Satisfaction	0.43^*^	0.5^**^	0.52^*^	−0.002	0.26	0.16	0.76^**^	0.02	0.71^**^	0.14

Abbreviations: FFMQ, Five Facet Mindfulness Questionnaire; FSFI, Female Sexual Function Index; KSS, Sexual Satisfaction Questionnaire; NSD, no sexual dysfunction; WSD, with sexual dysfunction.

a Correlation analysis based on *r* (Pearson) and rho (Spearman), depending on normal distribution of variables.

^*^ *P* < .05.

^**^ *P* < .01.

### Influence of mindfulness training on sexual functioning, sexual satisfaction, and the dimensions of mindfulness

We found a significant difference between groups in the dimensions of sexual response (Wilks lambda, *F*[18, 39] = 6.22; *P* < .001, η^2^ = 0.74). Out of 21 dependent variables, 4 did not differ between the WSD and NSD groups (*P* > .05): lubrication at posttraining, pain at posttraining, arousal at follow-up, and lubrication at follow-up.

**Figure 1:**
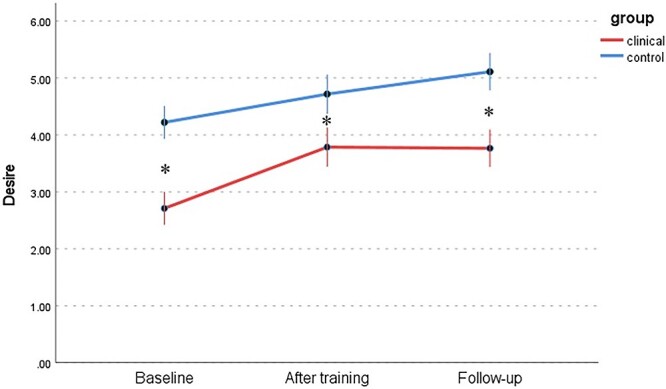
Changes in desire (FSFI) between the WSD (clinical) and NSD (control) groups at 3 time points. FSFI, Female Sexual Function Index; NSD, no sexual dysfunction; WSD, with sexual dysfunction.

**Figure 2:**
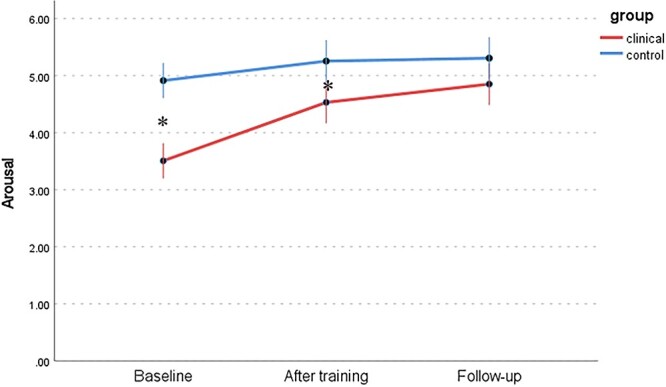
Changes in arousal (FSFI) between the WSD (clinical) and NSD (control) groups at 3 time points. FSFI, Female Sexual Function Index; NSD, no sexual dysfunction; WSD, with sexual dysfunction.

**Figure 3:**
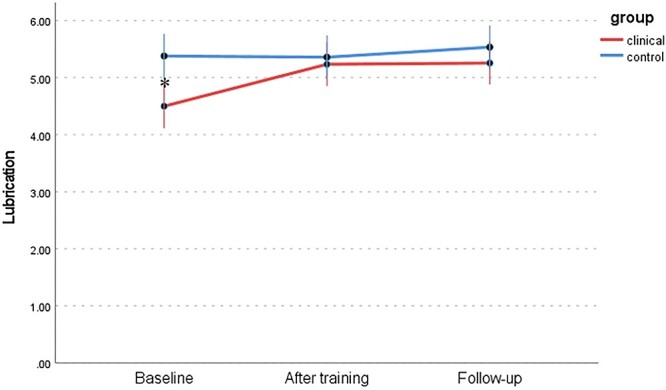
Changes in lubrication (FSFI) between the WSD (clinical) and NSD (control) groups at 3 time points. FSFI, Female Sexual Function Index; NSD, no sexual dysfunction; WSD, with sexual dysfunction.

**Figure 4:**
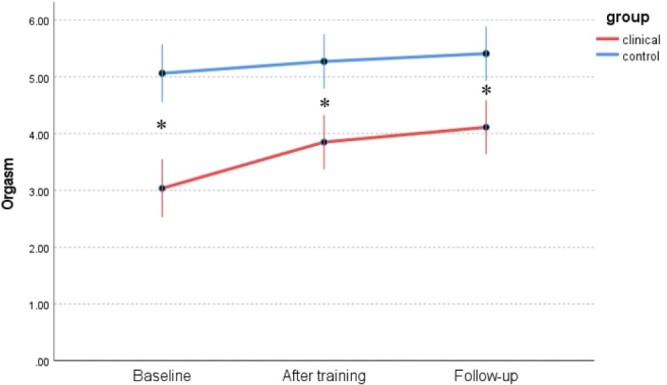
Changes in orgasm (FSFI) between the WSD (clinical) and NSD (control) groups at 3 time points. FSFI, Female Sexual Function Index; NSD, no sexual dysfunction; WSD, with sexual dysfunction.

**Figure 5:**
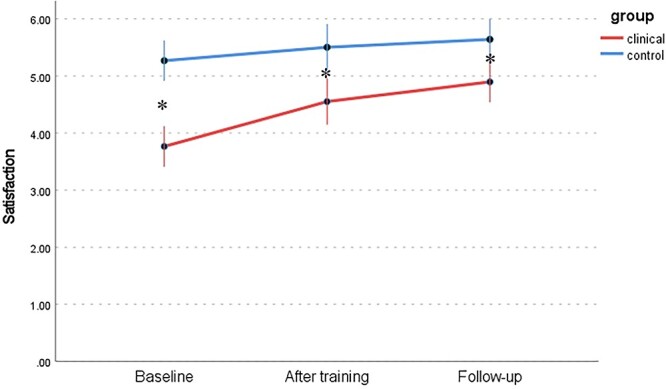
Changes in satisfaction (FSFI) between the WSD (clinical) and NSD (control) groups at 3 time points. FSFI, Female Sexual Function Index; NSD, no sexual dysfunction; WSD, with sexual dysfunction.

**Figure 6:**
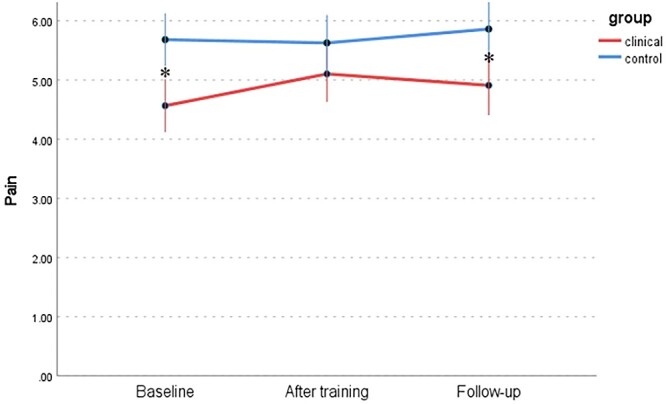
Changes in pain (FSFI) between the WSD (clinical) and NSD (control) groups at 3 time points. FSFI, Female Sexual Function Index; NSD, no sexual dysfunction; WSD, with sexual dysfunction.

**Figure 7:**
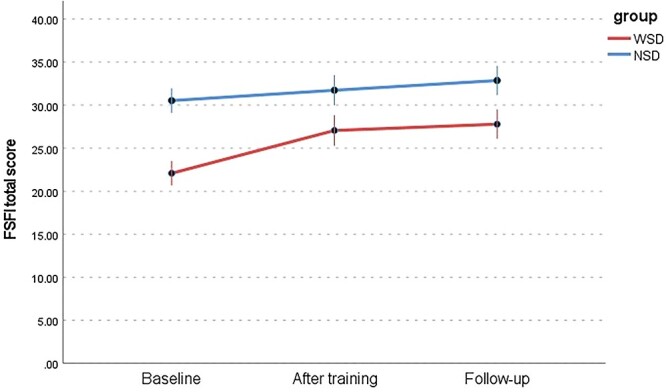
Changes in risk of sexual dysfunction (total FSFI score) in the WSD and NSD groups at 3 time points. FSFI, Female Sexual Function Index; NSD, no sexual dysfunction; WSD, with sexual dysfunction.

Changes in 6 dimensions of sexual response (FSFI) between the WSD and NSD groups at 3 time points are presented in Table 9 and Figures 1 to 6: desire, arousal, lubrication, orgasm, satisfaction, and pain. Changes in total score (FSFI), that idicates risk of sexual dysfunction, between the WSD and NSD groups at 3 time points are presented in Figure 7.

**Table 6:** Women who met cutoff scores for a high probability of sexual dysfunction (FSFI): baseline, 1 week after intervention, and follow-up.

		**WSD, %**			**NSD, %**		
**FSFI**	**Cutoff**	**Baseline (n = 53)**	**1 wk (n = 34)**	**Follow-up (n = 30)**	**Baseline (n = 40)**	**1 wk (n = 31)**	**Follow-up (n = 29)**
Desire	3.6	94.2	61.8	56.7	42.5	6.5	3.4
Arousal	3.9	76.5	32.4	20.0	20.0	3.2	3.4
Lubrication	5.1	68.6	35.3	27.6	46.2	30.0	28.6
Orgasm	3.6	70.6	46.9	36.7	24.3	0	0
Satisfaction	4.4	76.9	47.1	33.3	27.5	9.7	10.3
Pain	5.6	73.1	58.8	40.0	42.5	22.6	20.7
Total	27.5	90.6	58.8	46.7	32.5	6.5	6.9

Abbreviations: FSFI, Female Sexual Function Index; NSD, no sexual dysfunction; WSD, with sexual dysfunction.

**Table 7:** Effects of mindfulness training on measures of sexual response (FSFI), sexual quality of life (KSS), and FFMQ at baseline.^a^

	**Baseline, mean (SD)**		
**Measure**	**WSD (n = 53)**	**NSD (n = 40)**	**Range**
FSFI			
Desire^***^	2.6 (0.9)	4.01 (0.98)	1.2-6.0
Arousal^***^	3.21 (1.17)	4.41 (1.36)	0-6.0
Lubrication^**^	4.06 (1.65)	4.88 (1.47)	0-6.0
Orgasm^***^	2.62 (1.63)	4.44 (1.65)	0-6.0
Satisfaction^***^	3.55 (1.29)	4.76 (1.4)	0.8-6.0
Pain^**^	4.02 (1.92)	5.12 (1.68)	0-6.0
Total^***^	20.05 (5.93)	27.61 (7.2)	2.0-36.0
KSS^***^	21.62 (5.33)	31.68 (6.56)	10.0-40.0
FFMQ			
Nonreactivity^***^	2.22 (0.72)	2.7 (0.6)	1.0-5.0
Observation	3.57 (0.78)	3.66 (0.61)	1.0-5.0
Aware actions^**^	2.86 (0.6)	3.21 (0.5)	1.0-5.0
Description	3.27 (0.9)	3.47 (0.52)	1.0-5.0
Nonjudgmental^*^	3.03 (0.86)	3.43 (0.79)	1.0-5.0

Abbreviations: FFMQ, Five Facet Mindfulness Questionnaire; FSFI, Female Sexual Function Index; KSS, Sexual Satisfaction Questionnaire; NSD, no sexual dysfunction; WSD, with sexual dysfunction.

a Independent-samples *t*-test.

^*^ *P* < .05.

^**^ *P* < .01.

^***^ *P* < .001.

**Table 8:** Effects of mindfulness training on dimensions of sexual response (FSFI) at baseline, after training, and follow-up among women with a specific sexual dysfunction.

	**Group with a specific sexual dysfunction**			
**FSFI** ^**a**^	**Mean**	**SD**	**η** ^**2**^	***P* value**
Desire			0.46	<.001
Baseline	2.84	0.77		
After training	3.93	1.12		
Follow-up	4.06	1.24		
Arousal			0.38	<.001
Baseline	2.91	0.62		
After training	4.19	1.37		
Follow-up	4.56	1.44		
Lubrication			0.22	.002
Baseline	4.11	1.17		
After training	4.93	1.3		
Follow-up	4.97	1.31		
Orgasm			0.45	<.001
Baseline	1.9	0.61		
After training	3.28	1.33		
Follow-up	3.44	1.67		
Satisfaction			0.53	<.001
Baseline	3.41	0.77		
After training	4.43	1.25		
Follow-up	4.83	1.16		
Pain			—	.08
Baseline	4.25	1.39		

Abbreviation: FSFI, Female Sexual Function Index.

a With the exception of pain, each FSFI domain demonstrated intergroup significant differences with baseline (ie, baseline vs postintervention and baseline vs follow-up).

**Table 9:** Effects of mindfulness training on dimensions of sexual response (FSFI) at baseline, after training, and follow-up in the WSD group.

	**WSD**			
**FSFI** ^**a**^	**Mean**	**SD**	**η** ^**2**^	***P* value**
Desire			0.38	<.001
Baseline	2.64	0.77		
After training	3.73	1.09		
Follow-up	3.77	1.00		
Arousal			0.33	<.001
Baseline	2.92	0.63		
After training	4.23	1.39		
Follow-up	4.67	1.41		
Lubrication			0.19	.001
Baseline	3.74	1.3		
After training	4.84	1.55		
Follow-up	4.84	1.54		
Orgasm			0.23	<.001
Baseline	1.76	0.55		
After training	3.18	1.41		
Follow-up	3.29	1.78		
Satisfaction			0.37	<.001
Baseline	3.34	0.8		
After training	4.4	1.28		
Follow-up	4.82	1.17		
Pain			—	.39
Baseline	3.92	1.56		
After training	4.7	1.53		
Follow-up	4.78	1.89		

Abbreviations: FSFI, Female Sexual Function Index; WSD, with sexual dysfunction.

a With the exception of pain, each FSFI domain demonstrated intergroup significant differences with baseline (ie, baseline vs postintervention and baseline vs follow-up).

The overall risk for sexual dysfunction (FSFI) decreased from 90.6% at baseline to 46.7% at follow-up in the WSD group and from 32.5% at baseline to 6.9% at follow-up in the NSD group. Among the WSD group, the risk of sexual dysfunction was higher in the baseline measurement (mean = 22.08, SD = 4.4) as compared with the posttraining measurement (mean = 27.06, SD = 5.96) and follow-up (mean = 27.97, SD = 6.00; *F*[2, 56] = 14.77; *P* < .001, η^2^ = 0.35). In the control group, the risk of sexual dysfunction was significantly lower at the follow-up than at the baseline measurement.

#### Sexual quality of life: KSS

A significant increase in sex-related quality of life was observed among women in the WSD group (*F*[1.528, 42.797] = 34.55, *P* < .001, η^2^ = 0.55]. Women from the WSD group reported the lowest level of sexual satisfaction before the training (mean = 22.48, SD = 5.38) as compared with the posttraining measurement (mean = 27.9, SD = 5.16) and the follow-up (mean = 30.41, SD = 5.99). Additionally, sexual satisfaction was significantly (*P* < .001) higher in the follow-up measurement than in the posttraining measurement. In the NSD group, sexual satisfaction in the follow-up measurement was significantly higher than in the baseline measurement (*F*[2, 56] = 57.79, *P* < .001, η^2^ = 0.36].

#### Homework

The frequency of practicing meditation as homework was significantly related to a risk reduction in various sexual dysfunctions and an improvement in sex-related quality of life. We found significant differences between practicing and nonpracticing women across time (Tables 10 and 11).

## Discussion

To our best knowledge, this is the first study aimed at assessing the effectiveness of mindfulness monotherapy in sexual dysfunction treatment in women.^42^^,^^43^

### Diagnosis based on questionnaires and interviews

As predicted, participants in the WSD group started training with lower levels in particular domains of sexual functioning and sexual quality of life as compared with the NSD group. Some women from the NSD group obtained results on the FSFI indicating a risk of dysfunction at baseline, 1 week after training, and follow-up, as opposed to the assessment based on a standardized diagnostic interview conducted by the moderator. The absence of dysfunction, as assessed per *DSM-5* criteria, can be attributed to the absence of suffering and discomfort related to sexual activity and to the subjective assessment of satisfaction regarding sexual activities. In this context, the core of the *DSM-5* diagnostic criteria of sexual dysfunctions should be taken into account, in which attention is drawn to the need to reduce the probability of excessive and unnecessary diagnoses^66^ by specifying their presence based on at least 75% of the frequency of difficulties during sexual activity. During the interviews, the participants declared that they experienced specific difficulties (eg, pain, desire, difficulty in achieving orgasm) sporadically or sometimes and that these were not a source of discomfort. It is worth bearing in mind that the occasional occurrence of difficulties during sexual activity is a natural phenomenon and not a condition of the diagnosis and treatment. Additionally, the FSFI cannot be considered a stand-alone tool confirming the presence of sexual dysfunction, due to the lack of a scale that would separately assess the level of distress. The presence of subjectively experienced suffering is required to formulate a diagnosis in the *DSM-5*,^67^*ICD-10*,^68^ and *ICD-11*.^69^ In future research, the Female Sexual Distress Scale could also be used.^70^

### FSFI and KSS results

**Figure 8:**
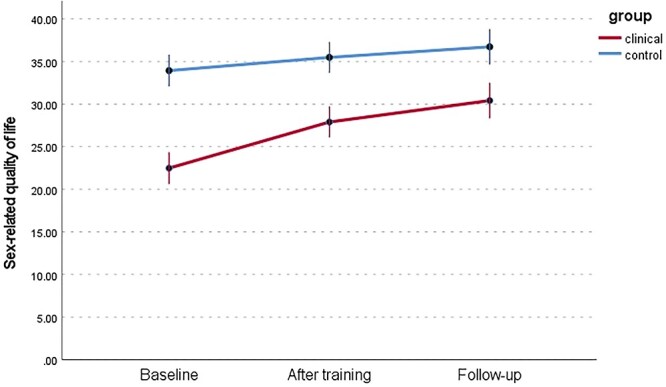
Changes in the sex-related quality of life (KSS) in the WSD and NSD groups at 3 time points. KSS, Sexual Satisfaction Questionnaire; NSD, no sexual dysfunction; WSD, with sexual dysfunction.

We hypothesized a significant improvement in sexual functioning among women participating in the mindfulness program and regularly performing the recommended mindfulness practices, as well as an improvement in sexual functioning attributed to an increase in subjective assessment of sexual satisfaction (sexual quality of life). It is worth discussing the differences in scores among the individual subscales. The arousal and lubrication scales are conceptualized differently: arousal as subjective arousal and lubrication as an element of genital response. An interesting difference in the results in domains mentioned above appears in the NSD group after the training and follow-up measurements (Figure 2 and Figure 3). The results indicate that the lack of consistency in this group persisted after the training. In the WSD group, the difference decreased significantly, which is in line with the results of previous mindfulness research and laboratory studies that examined the association between sexual concordance and interoception.^71^^,^^72^ A significant difference was observed between genital and subjective arousal in the NSD group, confirming the need for further research. At the same time, scores on the KSS were high (Figure 8). Despite the notable difference, the quality of sex life was not reduced in the NSD group. It is worth remembering that lubrication is not the only element of the genital response indicating arousal: apart from it, there is also increased sensitivity and swelling of the genital organs.^10^ In addition, lubrication can be explained by relatively common problems in women: age, the presence of sexually transmitted infections, or the use of hormonal contraception.^73^

In the NSD group, the participants obtained reasonably high scores on the pain scale, which remained relatively high. As previously mentioned, the FSFI cannot be used as an independent tool to assess the occurrence of sexual dysfunction, due to the absence of an assessment of distress. Moreover, the results in the pain domain are not recommended for diagnosis owing to the lack of established cutoff points for clinical groups.^66^ Additionally, these results are consistent with the results of other mindfulness studies in which no significant differences were noted in the pain domain.^42^^,^^44^ The aforementioned mindfulness studies also included cognitive-behavioral therapy and psychoeducation elements.

Similarly, in the WSD group, the desire subscale demonstrated low levels of improvement in measurements after the training and at follow-up. In the NSD group, despite the relatively high baseline scores, the participants improved significantly. At the same time, the WSD group initially declared a low quality of sexual life.

We can interpret these results in several ways. First of all, the decrease in or absence of sexual desire may be secondary to the presence of another sexual dysfunction, such as sexual pain or orgasmic disorders. Pain during sexual activity and lack of orgasm can increase frustration, fear of failure, or pain and lower mood.^74^ In addition, the participants initially achieved low results in the KSS, which measures not only one’s satisfaction with the relationship with a partner but also one’s subjectively assessed sexual attractiveness or thinking about oneself as a sexual partner. According to the circular model of female sexuality, the factors that reward and encourage one to engage in sexual activity or respond to stimuli are, among others, nonsexual factors such as deepening closeness and a harmonious relationship with a partner, as well as confirmation of one’s femininity and attractiveness in the partner’s eyes.^9^ A low-rated quality of sexual life, negative self-thoughts, and frustration with the relationship decrease a woman’s sexual activity and lower her motivation to be sexually active.^5^^,^^75^ Finally, sexual desire is related to the individual assessment of the attractiveness of the partner, the duration of the relationship, and the routine and habits that have developed over time.^76^ Given that many sexual issues result from problematic or unsatisfactory relationships, we can assume that some women experience sexual problems because of relationship difficulties.^77^ Therefore, an intervention developed for women only may not be sufficient, and it would seem reasonable to bring attention to the relationship between sexual problems and interpersonal difficulties.

Additionally, we analyzed the impact of mindfulness training on different domains of sexual response diagnosed with the FSFI. After selecting the groups of women in whom a given sexual dysfunction can be diagnosed, we checked how it changed under the influence of mindfulness training. The training proved effective, as we saw increases in all areas except pain. These results are consistent with the analysis of the entire WSD group without dividing it into specific sexual dysfunctions.

### F‌FMQ results

We examined the correlation between the individual dimensions of mindfulness measured by the FFMQ and the sexual satisfaction and FSFI domains. The results indicated a persistent strong correlation (1) between the nonreactivity and description dimensions and the improvement of functioning in various sexual areas and sex-related quality of life and (2) between the aware-actions and nonjudgmental dimensions and sex-related quality of life. *Nonreactivity* means low reactivity to stimuli reaching the field of attention, with a low threshold of emotional arousal. At the same time, *describe* signifies the ability to view the elements of experience passing through consciousness from a perspective, with the accompanying ability to label all present experiences. These results seem to be related to and consistent with the results of studies on cognitive and emotional factors influencing the occurrence and persistence of sexual dysfunctions (eg, automatic thoughts and external distractors) and emotional dissatisfaction.^78–80^ The results confirm that lowering the tendency to avoid attention and distraction under the influence of distractors contributes to improving sexual functioning. Additionally, cognitive distractors have been indicated as a strong predictor of problems with genital reactions among women.^49^^,^^81^ Therefore, it seems that the ability to focus on “here and now” and on body sensations may contribute to improving women’s sexual response in the form of lubrication. Similar conclusions apply to the orgasm and KSS domains, with the nonreactivity dimension correlated. According to previous research on cognitive factors involved in the development of orgasmic difficulties, the capability of experiencing orgasm is strongly related to the image of oneself and one’s body.^82^ We can assume that not identifying with negative thoughts arising during sexual activity and not engaging in automatic thoughts and emotions affects women’s more remarkable ability to experience orgasm and sexual satisfaction.

**Table 10:** Differences between women practicing and not practicing “homework” meditation in risk of sexual dysfunction (FSFI) and sex-related quality of life (KSS) by meditation type.^a^

	**Body scan**	**Long practice** **week 2**	**Mindful movement**	**Sexuality related**	**Mindful breathing**	**Mindful activity**
Risk of sexual dysfunction						
Arousal	↓P ↑NP^*^	↓P ↑NP^*^			↓P ↑NP^*^	
Lubrication	↓P ↑NP^*^					
Desire		↓P ↑NP^*^		↓P ↑NP^*^		
Satisfaction				↓P ↑NP^*^	↓P ↑NP^*^	
Orgasm						↓P ↑NP^*^
Sex-related quality of life			↑P ↓NP^*^	↑P ↓NP^***^		

Abbreviations: FSFI, Female Sexual Function Index; KSS, Sexual Satisfaction Questionnaire; NP, not practicing; P, practicing.

a Baseline vs second measurement.

^*^ *P* < .05.

^***^ *P* < .001.

**Table 11:** Differences between practitioners and nonpractitioners in risk of sexual dysfunction (FSFI) and sex-related quality of life (KSS) regarding by meditation type.^a^

	**Body scan**	**Long practice** **week 2**	**Long practice week 3**	**Long practice week 4**	**Sitting**	**Mindful movement**	**Sexuality related**
Risk of sexual dysfunction							
Arousal	↓P ↑NP^**^				↑P ↓NP^*^	↓P ↑NP^*^	↓P ↑NP^**^
Lubrication	↓P ↑NP^*^		↓P ↑NP^*^	↑P ↓NP^*^		↓P ↑NP^*^	↓P ↑NP^**^
Desire	↓P ↑NP^***^	↓P ↑NP^**^	↓P ↑NP^*^	↑P ↓NP^*^	↑P ↓NP^*^	↓P ↑NP^**^	
Satisfaction	↓P ↑NP^*^	↓P ↑NP^*^	↓P ↑NP^**^	↑P ↓NP^**^	↑P ↓NP^**^	↓P ↑NP^**^	↓P ↑NP^***^
Orgasm							
Pain			↓P ↑NP^**^			↓P ↑NP^**^	
Total	↓P ↑NP^*^	↓P ↑NP^**^	↓P ↑NP^**^	↑P ↓NP^*^	↑P ↓NP^**^	↓P ↑NP^**^	↓P ↑NP^***^
Sex-related quality of life	↑P ↓NP^*^	↑P ↓NP^**^	↑P ↓NP^*^	↑P ↓NP^**^	↑P ↓NP^**^	↑P ↓NP^*^	↑P ↓NP^***^

Abbreviations: FSFI, Female Sexual Function Index; KSS, Sexual Satisfaction Questionnaire; NP, nonpractitioner; P, practitioner.

a Baseline vs follow-up.

^*^ *P* < .05.

^**^ *P* < .01.

^***^ *P* < .001.

The aware-actions domain signifies acting with the awareness of what one does (eg, tasting food, washing dishes, dynamics of walking). Bringing mindfulness to daily activities is the opposite of operating on an “automatic remote control,” in which one pays no attention to undertaking various daily activities and tasks.^54^ The nonjudging domain signifies the nonassessment and nonjudgmental ability to observe thoughts, emotions, and bodily sensations appearing in the attention field, as opposed to blocking and repressing them, as associated with the ability to experience unpleasant sensations without ruminating. The results of recent studies that examined the association between mindfulness and sexual well-being show that an increase in awareness and presence during sexual activity contributes to greater satisfaction with sexual activity.^83^^,^^84^ This also seems consistent with the “good enough sex” model^85^ as well as with research on sensate focus, the exercises of which, when practiced, contributed to an increase in the sexual satisfaction of both partners.^24^

### Homework

We checked whether and how the home practice improves sexual functioning. We hypothesized (hypothesis 4) that the effects of mindfulness training in the form of improved sexual functioning and sexual satisfaction will persist 3 months after the end of the program to a greater extent among those who continue practicing the proposed meditation. As we predicted, a higher frequency improved sexual functions. The practice of mindful movement (*t*[14 710] = −3.18, *P* = .01) resulted in lower scores in the pain domain among women who practiced at home as compared with women who did not. The outcomes are in line with the intention of the exercise, which is the participant learning the boundaries of her body, responding to the boundaries that the body sets, and, at the same time, examining the so-called windows of tolerance (ie, one’s readiness to break these boundaries at a given moment or respect them).^86–88^ The goal of mindful yoga is therefore not to exert pressure on the body. The aim is to consciously direct attention and relaxation to painful, tense spots and simultaneously check how much one can do in a given moment despite the discomfort.^54^ In contrast, participants who practiced sexuality meditation reported a higher level of satisfaction with desire than women who practiced it rarely or not at all. According to previous reports on inhibitory control mechanisms in the regulation of sexual behavior, desire relates to the aforementioned factors: restrictive cultural norms and related inhibitions, as well as beliefs about oneself (eg, as a sexual partner).^89^ We can assume that, as a result of this practice, the participants gained more acceptance of themselves, their needs, and, as a result, the capability of their implementation, which is one of the effects of regular meditation.

### Dropout

Between the first and second measurements, we observed a dropout of participants in the WSD and NSD groups. While explaining the reasons for their resignation, the participants informed us about the time that needed to be devoted and the involvement in individual practice. We can conclude that therapy based on learning meditation is a form of work for motivated individuals. During the interview, the moderator talked with the participants about meditation and what it was, but it was impossible to assess the patients’ attitudes to the ideas related to it and the actual experience. Several analyses of mindfulness programs show that involvement in individual mindfulness practice between meetings varies: in some programs, >80% of participants in a given group regularly engage in homework; in others, about 30% to 40% and even 14%.^90–92^

### Methodological diversity

The research methodology on the effectiveness of mindfulness practices in the treatment of sexual dysfunctions to date is diverse. The number of proposed stationary meetings and their duration differ; nevertheless, the results of the studies indicate the effectiveness of the proposed interventions, regardless of the number of meetings, their duration, and the proposed practices.^44^ It is no less difficult to assess the effectiveness of specific mindfulness practices, considering the inclusion of other therapeutic techniques in clinical interventions. This study is the first to use mindfulness as monotherapy. Previously, it was proposed to standardize the structures of therapy for sexual dysfunctions,^42^ based primarily on other mindfulness-based programs (MBSR, mindfulness-based cognitive therapy, mindfulness-based compassionate living, mindfulness-based relapse prevention).^93^ Based on our preliminary results, it seems that separate programs should be developed for specific sexual dysfunctions, with an emphasis on pain disorders.

### Strengths and limitations

This study is the first investigation to check how mindfulness monotherapy contributes to the reduction of symptoms of sexual dysfunction and the improvement of sex-related quality of life. The study used validated questionnaires treated as the gold standard in the assessment of sexual function (FSFI)^94^ and mindfulness indicators (FFMQ).^95^ The study design included a battery of questionnaires assessing sexual functioning as best as possible, which made it possible to obtain initial explanations regarding factors lowering the risk of sexual dysfunction. Another advantage of the study is the analysis of individual practices between meetings and assessment of their effectiveness. Moreover, this is the first study to focus only on psychogenic sexual dysfunctions. We conducted the preliminary interviews with participants with attention to diseases, disorders, and medications that could affect the sexual response and interfere with the objectivity of the results.^42^ The advantage of the procedure is also the fact that the training was moderated by a mindfulness teacher who has well-established individual practice, thereby fostering quality assistance and understanding of the participants’ experiences. The study’s strength is a comparison of the influence of mindfulness techniques in the group of women with and without sexual dysfunctions.

Separate attention is needed to consider the internal and external validity of the research findings. One of the limitations worth mentioning is that the training sessions were provided by one of the authors, who also designed the study. Unintentionally though, the researcher might have influenced participants’ behavior by, for example, emphasizing the significance of the study and being more or less informative toward various groups. However, the intervention protocol was meticulously implemented to avoid any difference in therapeutic approach between the groups. Second, the study group (help-seeking women with sexual dysfunction) may not be representative of the entire population of women with sexual dysfunctions. The study participants did not reflect the diversity of the general population and were cisgender, heterosexual, and predominantly educated and employed, which may limit the generalizability of the findings to populations with different sexual identities, values, behaviors, and messages around sexuality. Also, several women withdrew or did not show up without explanation; therefore, it is difficult to comment on the characteristics of the whole sample. Moreover, the study included women with psychogenic sexual dysfunction who were interested in learning how to meditate (ie, motivated and willing to learn) and who had responded primarily to social media advertisements. It is unknown whether our results would generalize to the larger population of women with sexual dysfunction, as those who participated may have been more motivated. The major limitations of the study are the lack of a double-blind procedure, randomization, an appropriate control group subjected to a different therapeutic method of proven effectiveness, and the formation of a group of participants with dysfunctions who are on a waiting list for therapy. We were not able to understand and evaluate the potential placebo effect, and in the absence of a control group, our results must be interpreted with great caution. Without the randomization to treatment and a double-blind procedure, we cannot reduce the likelihood that any observed beneficial effects occurred due to factors outside the experimental treatment. The applied mindfulness training may have been beneficial for several reasons (eg, group support, cost-effectiveness as opposed to individual long-term therapy); therefore, it is difficult to form precise conclusions. Future efficacy testing will require a comparison group to rule out nonspecific factors as alternative causes of improvement. Future research is needed to evaluate these interventions for women in same-sex relationships and single women. In our study, we used the FSFI, which cannot be considered a tool confirming the presence of sexual dysfunction. Thus, we decided to assess the level of sexual distress during the clinical interview with a sexologist. However, in future research and for a better clarity and study aim, it could be necessary to use a scale dedicated solely to sexual distress, such as the Female Sexual Distress Scale.^96^

### Possible application in clinical practice

The study results have a chance to translate to (1) an introduction of a new therapeutic program for specialists, (2) the provision of more effective help to women experiencing sexual difficulties or an inability to undertake sexual activity, and (3) an indication of the deterioration in sexual quality of life. Due to their clinical usefulness, mindfulness practices can be a valuable supplement to sex therapy specialist tools.

## Conclusion

The applied mindfulness training was beneficial in the treatment of sexual dysfunctions in terms of increasing desire and arousal, as well as the ability to reach orgasm. An increase in sexual quality of life was also observed after the intervention. However, this therapeutic approach certainly needs more investigation before it can be recommended as an effective intervention in the treatment of sexual dysfunction. In particular, the studies on mindfulness-based therapy should be replicated on larger and diverse samples of patients with sexual dysfunctions, including men, and under a more rigorous research design, including adequate control groups and random allocation of participants to study conditions. The presented therapy model is safe for and well tolerated by patients. Patients can benefit from the therapy program if they are motivated and ready to learn how to meditate, devote a lot of time to individual practice, and tolerate the frustration of tedious daily practice and exercises. Repetition of individual practice can improve contact with the body, increase the ability to observe emerging sensations (including those during sexual activity), reduce the presence of automatic thoughts and distractors, and raise the concentration on the “here and now” during sexual activity.

## Funding

None declared.

*Conflicts of interest:* Authors have no conflict of interest to declare.

## Supplementary Material

Suplementary_materials_with_tables_qfad022
