# Supplementary material for: Assessment of the effect of mindfulness monotherapy on sexual dysfunction symptoms and sex-related quality of life in women
Source: Sex Med. 2023 Jun 5;11(3):qfad022. doi: 10.1093/sexmed/qfad022 (PMC10243933; doi:10.1093/sexmed/qfad022)
Supplement: Suplementary_materials_with_tables_qfad022 [file suplementary_materials_with_tables_qfad022.docx]

Supplementary materials

Table S1. Description of the program week-by-week

*Insert table S1*

Detailed results

KSS - WSD

We observed a significant main effect of measurement time [F(2, 112)=49.45, p<0.001, eta^2^=0.47], and the main effect of the group [F(1,56)=48.85, p<0.001, eta^2^=0.47]. The effect of time and group interaction was also significant [F(2, 112=11.95, p<0.001, eta^2^=0.18]. There was an increase in sexual satisfaction in the whole group, in comparison to the measurement before the training (M=28.21, SD=7.63) to the post-training measurement (M=31.69, SD=6.18) and the follow-up measurement (M=33.57, SD=6.42). The level of sexual satisfaction was significantly higher in the follow-up measurement compared to the post-training measurement. Further post hoc tests were carried out, which showed that in all 3 measurements, the WSD group obtained significantly lower scores on the level of sexual satisfaction than the NSD group. In the WSD group, the level of sexual satisfaction was the highest in the follow-up measurement, and significantly different from the post-training measurement, and the baseline.

FSFI - Desire

We observed a significant main effect of measurement time [F(2, 112)=35.79, p<0.001, eta^2^=0.39] and the main effect of the group [F (1.56)=50.23, p <0.001, eta^2^=0.47]. The effects of time and group interaction turned out to be insignificant (p=0.054). In the whole group, there was an improvement in the area of ​​desire, in the comparison to the measurement before the training (M=3.47, SD=1.08) to the measurement after training (M=4.25, SD=1.03) and to the follow-up measurement (M=4.44, SD=1.11).

FSFI - Arousal

We observed a significant main effect of measurement time [F (2, 112)=20.11, p <0.001, eta2=0.26], and the main effect of the group [F(1,56)=22.2, p<0.001, eta^2^=0.28]. The effect of time and group interaction was also significant [F(2, 112=5.78, p=0.004, eta^2^=0.09]. In the whole group, there was an increase in satisfaction with arousal, in the comparison of the measurement before training (M=4.21, SD=1.09) to the measurement after training (M=4.89, SD=1.05) and the follow-up measurement (M=5.08, SD=1.01).

Lubrication - FSFI

We have observed a significant main effect of measurement time [F(2, 112)=3.33, p=0.002, eta^2^=0.1] and an effect of time and group interaction [F(2, 112=4.44, p<0.02, eta^2^=0.07]. group turned out to be insignificant (p=0.06). In the whole group, there was an increase in satisfaction with the level of lubrication, in the comparison of the measurement before training (M=4.94, SD=1.13) to the measurement after training (M=5.3, SD=1.02) and the follow-up measurement. (M=5.39, SD=1.01).

Orgasm - FSFI

We observed a significant main effect of measurement time [F(2, 112)=10.85, p<0.001, eta^2^=0.16] and the main effect of the group [F(1,56)=28.96, p<0.001, eta^2^=0.34]. The effect of time and group interaction also appeared to be significant [F(2, 112=3.09, p=0.049, eta^2^=0.05]. There was an increase in orgasm satisfaction in the whole group, in the comparison of the measurement before training (M=4.05, SD=1.7) to measurement after training (M=4.56, SD=1.47) to the follow-up measurement (M=4.76, SD=1.43).

Satisfaction - FSFI

We observed a significant main effect of measurement time [F(2, 112)=18.53, p<0.001, eta^2^=0.25], and the main effect of the group [F(1,56)=23.26, p<0.001, eta^2^=0.29]. The effect of time and group interaction was also significant [F(2, 112=4.84, p=0.01, eta^2^=0.08]. There was an increase in satisfaction with sexual activity in the whole group, in the comparison of the measurement before the training (M=4.52, SD=1.22) to the post-training measurement (M=5.03, SD=0.97) and the follow-up measurement (M=5.27, SD=1.03).

Risk of sexual dysfunction - FSFI

We observed a significant main effect of measurement time [F(2, 112)=24.12, p<0.001, eta^2^=0.3] and the main effect of the group [F(2, 112=5.84, p=0.004, eta^2^=0.09]. The effect of time and group interaction was also significant [F(2, 112=5.84, p=0.004, eta^2^=0.09]. There was a decrease in the risk of sexual dysfunction in the whole group, in the comparison of the measurement before the training (M=26.31, SD=5.72) to after the training (M=29.39, SD=5.3) and follow-up measurement (M=30.33, SD=5.22).

**Homework**

The NSD group

In Measure 2, the analysis of the effect of homework frequency on sexual functioning and satisfaction showed the following: *sitting meditation* practice (*t*(29) = -2.15; *p*=0.04). The women who practiced *sitting meditation* reported a higher level of sexual satisfaction (*M*=36.12, *SD*=4.5) than women who practiced it rarely or not at all (*M*=31.67, *SD*=4.76). *Long meditation practice (any)* (*t*(5.248) = -3.08; *p*=0.03). The practicing women reported a higher level of sexual satisfaction (*M*=36.92, *SD*=2.16) than women who practiced rarely or not at all (*M*=28.33, *SD*=6.74). *Meditation on sexuality* practice (*t*(5.303) = -3.12; *p*=0.02). The women who practiced meditation on sexuality were less at risk of sexual dysfunction (*M*=32.85, *SD*=1.52) than women who practiced it rarely or not at all (*M*=27.28, *SD*=4.31). *Attentive eating* practice (*t*(29) = -2.39; *p*=0.02). The practicing women were less at risk of sexual dysfunction (*M*=32.19, *SD*=2.65) than women who practiced it rarely or not at all (*M*=27.93, *SD*=5.5).

In Measure 3, the analysis of the effect of homework frequency on sexual functioning and satisfaction showed the following: *Long practice (any*(*t*(27) = -2.06; *p*=0.049). The practicing women were less at risk of sexual dysfunction (*M*=33.37, *SD*=2.31) than women who practiced rarely or not at all (*M*=31.27, *SD*=2.49). *Sitting meditation* practice (*t*(9.902) = -3.45; *p*=0.006). The practicing women were less at risk of sexual dysfunction (*M*=34.03, *SD*=0.93) than women who practiced it rarely or not at all (*M*=30.65, *SD*=3.02). *Sexuality meditation practice 4^th^ week* (*t*(4.265) = -3.34; *p*=0.03). The practicing women were less at risk of sexual dysfunction (*M*=33.69, *SD*=1.26) than women who practiced rarely or not at all (*M*=28.88, *SD*=3.17). *Attentive activity meditation* practice (*t*(27) = -2.08; *p*=0.047). The practicing women were less at risk of sexual dysfunction (*M*=33.28, *SD*=2.22) than women who practiced rarely or not at all (*M*=30.88, *SD*=2.91).

Table S2 shows the percentage of women who chose a specific practice in the last four weeks, broken down by the clinical and the study group at the 2nd and 3rd measurement

Table S2. Percentage of women practicing different types of meditation at least 2 times a week, divided into groups (WSD and NSD) after the training and at follow-up

| Table S1. Description of the program week-by-week | | |
| --- | --- | --- |
| Weeks | Content of the meeting | Individual practice (homework) |
| 1 Week | - A brief overview of the influence of psychogenic causes on the development and persistence of sexual dysfunctions  - "Raisin Exercise" with an overview of how to experience mindfulness and how to do what you do attentively  - "body scan meditation" with an overview of the experience  - mindful breathing, discussion  - a few minutes of meditation practice at the end of the meeting | - at least 3-4 times a week "body scan" meditation (45 minutes) with a recording  - information about the time of practice, the possibilities of space organization, discussed with the participants  - every day - one activity is performed attentively - e.g. washing hands, washing dishes, preparing and drinking coffee or tea (including e.g. pouring, smelling, observing emotional, mental, and physiological experiences while drinking)  - daily for approx. 3 minutes - conscious breathing (taking into account the observation of chest movement, casting the anchor of attention, listening to the rhythm of the breathing, not regulating it)  - the practice of observing pleasant experiences recorded in the diary to be discussed at the next meeting |
| Week 2 | - A minute of silence at the beginning of the meeting  - discussion of experiences with different practices last week: difficulties, challenges, reactions from the body, possible changes  - discussing the practice of pleasure (paying attention to pleasure, indulging in pleasure, reactions flowing from the body when recognizing pleasure)  - "mindful yoga" meditation with discussion  - conscious breathing  - a few minutes of meditation practice at the end of the meeting | at least 3-4 times a week "mindful yoga" meditation (45 minutes) with a recording - information about the time of practice and the possibilities of space organization, discussed with the participants  - every day - one activity done attentively  - every day - eating one meal attentively  - daily for approx. 3-5 minutes - conscious breathing  - the practice of observing unpleasant experiences recorded in the diary to be discussed at the next meeting  - everyday - observation of what makes it difficult to organize time for oneself, the number of activities performed consciously |
| Week 3 | - A minute of silence at the beginning of the meeting  - discussion of experiences with different practices last week: difficulties, challenges, reactions from the body, possible changes  - discussing the practice of unpleasantness (paying attention to pleasure, indulging in pleasure, body reactions when recognizing pleasure)  - "sitting meditation" with discussion  - "interoception" meditation with discussion  - a few minutes of meditation practice at the end of the meeting | - at least 4 times a week "sitting meditation" meditation (45 minutes) alternating with "interoception" meditation (30 minutes) with a recording  - every day - one activity done attentively  - every day - eating one meal attentively  - daily for approx. 3-5 minutes - conscious breathing  - on the received sheet with the outline of a figure, marking the reaction of areas of the body to e.g. desire, excitement, and observing whether they are pleasant or unpleasant, what emotions, thoughts, and experiences they cause |
| Week 4 | - A minute of silence at the beginning of the meeting  - discussion of experiences with different practices in the previous week: difficulties, challenges, reactions from the body, possible changes  - discussion of the body observation practice  - "sexuality" meditation with discussion  - "mountain meditation" with an overview  - summary, acknowledgements  - a few minutes of meditation practice at the end of the meeting | - at least 4 times a week "on sexuality" meditation (40 minutes) alternating with another main, long meditation with recording  - every day - one activity done attentively  - every day - eating one meal attentively  - daily for 3-5 minutes - conscious breathing |

Table S2. Percentage of women practicing different types of meditation at least 2 times a week, divided into groups (WSD and NSD) after the training and at follow-up

|  | After training | | Follow-up | |
| --- | --- | --- | --- | --- |
|  | WSD group  (*n*=34) | NSD group  (*n*=31) | WSD group  (*n*=30) | NSD group  (*n*=29) |
| Body scan – I week | 88.2 | 74.2 | 53.3 | 89.7 |
| Long practice - II week | 88.2 | 74.2 | 60.0 | 82.8 |
| Mindful yoga – II week | 58.8 | 54.8 | 50.0 | 51.7 |
| Long practice – interoception – III week | 76.5 | 77.4 | 63.3 | 75.9 |
| Sitting meditation – III week | 67.6 | 80.6 | 46.7 | 65.6 |
| Long practice - IV week | 50.0 | 83.9 | 43.4 | 79.3 |
| Sexuality meditation – IV week | 32.4 | 80.6 | 30.0 | 82.8 |
| Mindful breathing | 88.2 | 100 | 73.3 | 86.2 |
| Mindful eating | 76.5 | 90.3 | 53.3 | 72.4 |
| Mindful activity | 82.4 | 93.5 | 66.7 | 82.8 |
